# Epitope-based labeling for improved live-imaging of endogenous proteins in *C. elegans*

**DOI:** 10.1093/g3journal/jkag212

**Published:** 2026-08-31

**Authors:** Elise van der Salm, Mette H Schroeder, Loes B Steller, Stephanie I Miller, Amelie Scheper, Gwen Nowee, Erik E Griffin, Suzan Ruijtenberg

**Affiliations:** Division of Developmental Biology, Institute of Biodynamics and Biocomplexity, Department of Biology, Faculty of Science, Utrecht University, Padualaan 8, Utrecht 3584 CH, The Netherlands; Division of Developmental Biology, Institute of Biodynamics and Biocomplexity, Department of Biology, Faculty of Science, Utrecht University, Padualaan 8, Utrecht 3584 CH, The Netherlands; Division of Developmental Biology, Institute of Biodynamics and Biocomplexity, Department of Biology, Faculty of Science, Utrecht University, Padualaan 8, Utrecht 3584 CH, The Netherlands; Department of Biological Sciences, Dartmouth College, Hanover, NH 03755, United States; Division of Developmental Biology, Institute of Biodynamics and Biocomplexity, Department of Biology, Faculty of Science, Utrecht University, Padualaan 8, Utrecht 3584 CH, The Netherlands; Division of Developmental Biology, Institute of Biodynamics and Biocomplexity, Department of Biology, Faculty of Science, Utrecht University, Padualaan 8, Utrecht 3584 CH, The Netherlands; Department of Biological Sciences, Dartmouth College, Hanover, NH 03755, United States; School of Life Sciences, University of Warwick, Coventry CV4 7AL, United Kingdom; Division of Developmental Biology, Institute of Biodynamics and Biocomplexity, Department of Biology, Faculty of Science, Utrecht University, Padualaan 8, Utrecht 3584 CH, The Netherlands

**Keywords:** epitope-based labeling, SunTag, Frankenbody, MoonTag, AlfaTag, *C. elegans*, live imaging, microscopy, development

## Abstract

Visualizing protein expression dynamics with high temporal resolution is essential for understanding how cells acquire specific fates and functions during development, where key decisions can occur within minutes. Conventional direct fluorescent tagging often fails to capture these rapid changes in protein expression due to the relatively slow fluorophore maturation time. Indirect epitope-based labeling strategies offer a promising alternative, yet only a limited number of these systems have been developed and used in the context of multicellular organisms. Here, we evaluate and combine 4 epitope-based indirect labeling systems for live-imaging of proteins in *Caenorhabditis elegan*s: the SunTag, Frankenbody, MoonTag, and AlfaTag systems. Each system uses a fluorescently labeled high-affinity single-chain antibody or nanobody to recognize short peptide epitopes fused to a protein of interest, enabling immediate labeling of newly synthesized proteins. We demonstrate that all 4 systems specifically label epitope-tagged endogenous proteins and show no detectable cross-reactivity when used in dual-color combinations, enabling simultaneous visualization of distinct proteins within the same embryo. In addition, we show that the SunTag system offers 3 major advantages over direct labeling: earlier detection of proteins and enhanced sensitivity through signal amplification (as illustrated by CAM-1) and less impact on the function (as demonstrated for ERM-1). Together, this work provides a resource for the *C. elegans* community by establishing 4 orthogonal epitope-based labeling systems for multiplex live-imaging of endogenous proteins, opening new opportunities to study rapid protein dynamics during development.

## Introduction

Precise regulation of protein expression is essential for development and homeostasis of multicellular organisms, including *Caenorhabditis elegans*. During development, cells make rapid fate decisions that depend on the abundance, location, and activity of specific proteins. These changes in protein level can occur on timescales of minutes and can differ across cell types even within the same organismal environment (Sivaramakrishnan et al. 2023; Shukla et al. 2025). Measuring and visualizing these rapid changes in protein abundance and activity is therefore essential for understanding the mechanisms underlying rapid cell fate acquisition during development. Capturing such dynamics requires live-imaging approaches with high temporal resolution that allow us to measure protein levels and subcellular localization. In most standard live-imaging approaches, a fluorescent protein is directly fused to the protein of interest (POI) to visualize its expression. However, direct fluorescent tagging often fails to detect rapid changes in expression because the maturation time of fluorescent proteins is often longer than the window of protein expression. As a result, newly synthesized proteins cannot be observed immediately after translation, and rapid and dynamic changes in protein expression may be missed (Shukla et al. 2025). In addition, the relatively large size of fluorescent proteins (∼25 kDa) can interfere with protein function or localization, making direct tagging unsuitable for some developmentally important proteins (Bergwell et al. 2019).

To overcome these limitations, several epitope-based labeling systems have been developed, including SunTag, Frankenbody, MoonTag, and AlfaTag (Fig. 1a–d) (Tanenbaum et al. 2014; Boersma et al. 2019; Zhao et al. 2019; Akhuli et al. 2022). These indirect labeling systems use a single-chain variable antibody fragment (scFv) or nanobody fused to a fluorescent protein that binds with high affinity to short epitopes fused to the POI. Because the fluorescently labeled scFv or nanobody is already matured before binding to the epitope, POIs become fluorescently labeled immediately upon synthesis of the fused epitope. This enables real-time visualization of nascent proteins and visualization of short-lived protein dynamics that cannot be observed with direct fluorescent tags. Furthermore, the small size of the epitopes minimizes perturbation of protein function and allows for signal amplification when multiple epitopes are incorporated (Virant et al. 2018; Bergwell et al. 2019). Moreover, incorporating an array of peptides allows long-term tracking of translation dynamics of single mRNAs and has been instrumental in many key discoveries in translation regulation (Morisaki et al. 2016; Pichon et al. 2016; Wang et al. 2016; Wu et al. 2016; Yan et al. 2016). Beyond live-imaging applications, epitope-based systems can be adapted for a wide range of purposes, as scFvs or nanobodies can be fused not only to fluorophores but also to various effector proteins. Such applications enable, for example, protein purification (Götzke et al. 2019), transcriptional regulation at endogenous loci (Tanenbaum et al. 2014) and targeted DNA epigenome editing (Huang et al. 2017).

**Fig. 1.**
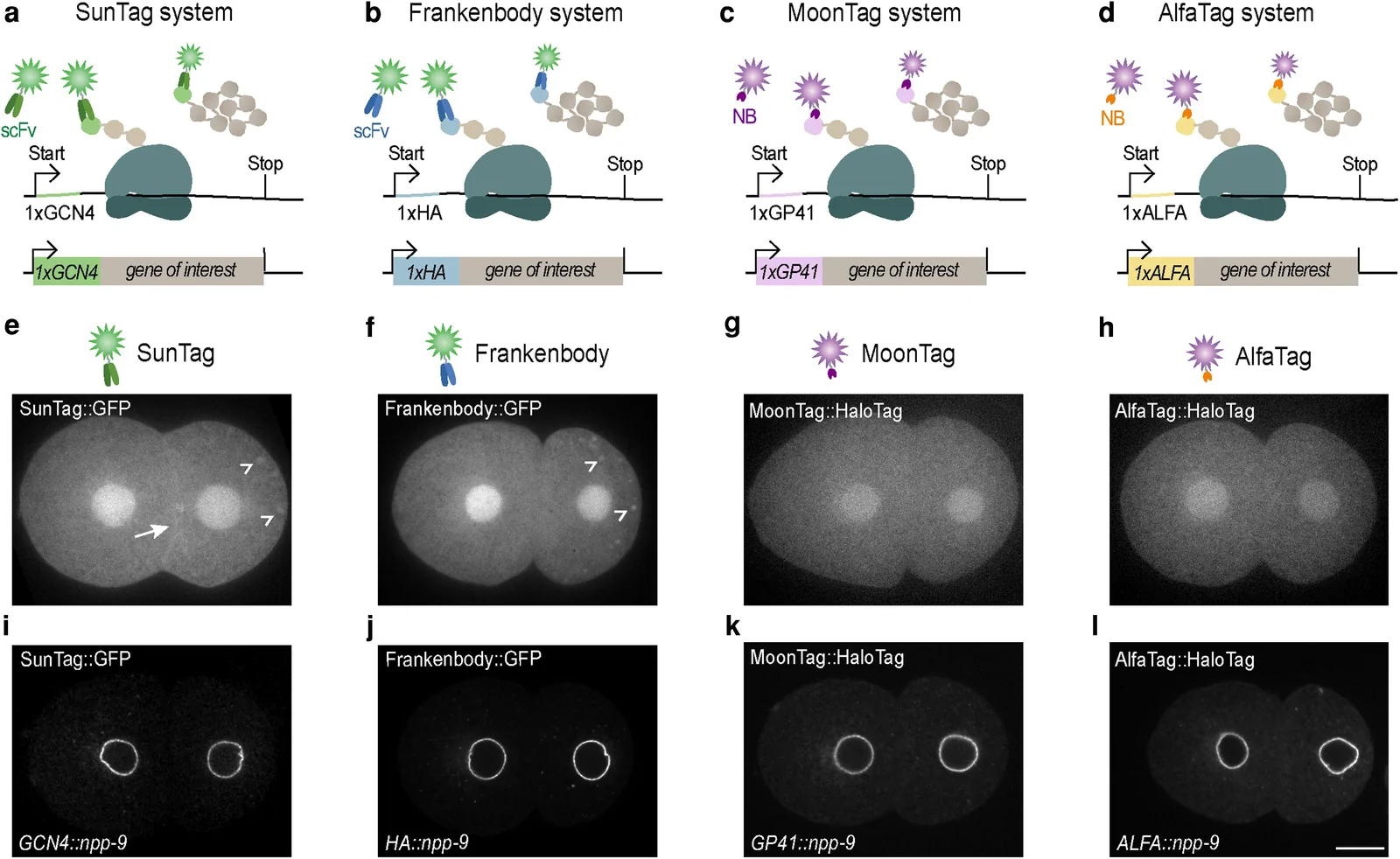
Schematic overview of the SunTag, Frankenbody, MoonTag, and AlfaTag fluorescent protein labeling systems. a–d) Schematics of 4 epitope-based labeling systems. SunTag and Frankenbody systems consist of a fluorescently labeled scFv that binds GCN4 (a) or HA (b) epitopes, respectively. MoonTag and AlfaTag systems consist of a fluorescently labeled nanobody (NB) that binds GP41 (c) or ALFA (d) epitopes, respectively. Binding occurs immediately upon synthesis of the epitope-tagged protein, enabling rapid visualization of the tagged POI. In this study, SunTag and Frankenbody scFvs were fused to GFP (green), whereas MoonTag and AlfaTag were fused to HaloTag (purple), allowing combinations of systems for simultaneous labeling of multiple proteins. e–h) Representative images of 2-cell stage *C. elegans* embryos expressing SunTag::GFP (e), Frankenbody::GFP (f), MoonTag::HaloTag (g), or AlfaTag::HaloTag (h). Arrow indicates enrichment at the membrane, open arrowheads indicate enrichment in granular structures. i–l) Representative images of NPP-9 labeling in 2-cell stage *C. elegans* embryos expressing SunTag::GFP and GCN4::NPP-9 (i), Frankenbody::GFP and HA::NPP-9 (j), MoonTag::HaloTag and GP41::NPP-9 (k), or AlfaTag::HaloTag and ALFA::NPP-9 (l). Different brightness/contrast settings have been applied. Scale bar: 10 μm.

Although epitope-based labeling systems are widely used in cell culture, only a limited subset has been adapted for use in multicellular model organisms (Zhao et al. 2019; Dufourt et al. 2021; Vinter et al. 2021; Murakawa et al. 2022; Xu et al. 2022; Hu et al. 2023; Boswell et al. 2025; Quintin et al. 2025; van der Salm et al. 2025a, 2025b; Jiang et al. 2026). Yet, such tools are especially valuable in multicellular organisms, where gene expression and protein dynamics can change rapidly during development. In *C. elegans,* for example, embryonic cells divide approximately every 20 min, requiring protein dynamics to be tightly regulated on similarly short timescales to ensure proper cell fate acquisition (Sivaramakrishnan et al. 2023; Shukla et al. 2025). Nevertheless, only SunTag and AlfaTag have been used in *C. elegans* for live-imaging of mRNA, translation, or proteins (Hu et al. 2023; Quintin et al. 2025; van der Salm et al. 2025a, 2025b). For most other systems, including the Frankenbody and MoonTag systems, it is unclear whether they can be used in *C. elegans*. Moreover, in contrast to cellular systems (Vigano et al. 2021), it has not been tested whether multiple epitope-based labeling systems can be combined in *C. elegans* without cross-reactivity to visualize multiple proteins simultaneously. The limited development and systematic validation of epitope-based labeling strategies in multicellular model organisms constrain our understanding of complex biological processes in a developmental context.

Here, we evaluate 4 epitope-based labeling systems, SunTag, Frankenbody, MoonTag, and AlfaTag, for live-imaging of proteins in *C. elegans* embryos and larvae. All 4 systems use high-affinity scFvs or nanobodies to recognize short peptide epitopes. The SunTag and Frankenbody systems consist of an scFv that binds to either GCN4 or HA epitopes, respectively. The MoonTag and AlfaTag systems consist of a nanobody that binds with high affinity to either GP41 or ALFA epitopes, respectively (Fig. 1a–d). We show that all 4 systems can be successfully applied in *C. elegans* to label POI in vivo, providing a versatile toolkit for live-imaging applications. By combining GFP-labeled scFvs (named SunTag::GFP and Frankenbody::GFP throughout the paper) with Halo-labeled nanobodies (named AlfaTag::HaloTag and MoonTag::HaloTag throughout the paper), we show that different proteins can be visualized simultaneously without detectable cross-reactivity. Furthermore, using the SunTag system we show that epitope-based labeling systems enable more rapid detection of proteins and have a lower impact on protein function than direct fluorophore fusions. Together, this work provides a resource for the *C. elegans* community by establishing and benchmarking 4 orthogonal epitope-tagging systems for multiplex protein imaging in vivo, opening new opportunities for real-time visualization of rapid protein dynamics during development.

## Methods

### *C. elegans* strains and maintenance

All strain names and associated genotypes can be found in Supplementary Table 1. Worm strains were cultured following standard procedures (Brenner 1974). Worms were grown on nematode growth medium (NGM) agar plates seeded with OP50  *Escherichia coli* (*E. coli*) at 15 °C or 20 °C.

### Strain generation

All *C. elegans* strains described in this study were generated using CRISPR/Cas9 for single-copy integration of transgenes; a detailed overview of all transgenes, including repair templates used and sgRNAs/crRNAs, is provided in Supplementary Table 2. For single-copy integrations at MosSCI loci, a plasmid-based CRISPR/Cas9 approach was used (Friedland et al. 2013). Injection mixes containing *eft-3p::Cas9* plasmid (50 ng/μL Addgene plasmid #46168), sgRNA plasmid (50 ng/μL), repair template plasmid (50 ng/μL), and co-injection marker plasmid (2.5 ng/μL) were delivered by microinjection into the gonads of young adult animals. Offspring expressing the co-injection marker were grown, lysed, and genotyped to select for genome-edited animals. All other single-copy insertions were performed using a Cas9 ribonucleoprotein-based method (Ghanta et al. 2021). Injection mixes contained Cas9 protein (0.25 μg/μL) (Integrated DNA Technologies [IDT]), tracrRNA (0.1 μg/μL) (IDT), pRF4 [rol-6(su1006)] (40 ng/μL) co-injection marker (Mello et al. 1991), locus-specific crRNA (56 ng/μL) (IDT) and either ssODN (0.11 μg/μL) (IDT) or melted dsDNA (25 ng/μL) repair template, or 100 ng/μL of plasmid pSM63 (GFP::Frankenbody repair template). dsDNA repair templates were synthesized by PCR using 5′ SP9 modified primers (IDT) and purified using the NucleoSpin Gel and PCR Clean-up Kit (Macherey-Nagel, 740609.250). For strains generated by crossing, hermaphrodites were heat-shocked at 30 °C for 6 h and recovered at 20 °C for 3 d to obtain males. Males were crossed with relevant strains, and progeny were genotyped.

### HaloTag labeling

HaloTag labeling was performed as previously described, by culturing animals in S-medium provided with OP50 and HaloTag ligand (Chang and Dickinson 2022; Borchers et al. 2025). In short, to prepare S-medium, autoclaved S-basal [NaCl (5.844 g/L), K_2_HPO_4_*3H_2_O (11.4115 g/L), and KH_2_PO_4_ (6.8045 g/L)] was provided with 1% (vol/vol) autoclaved trace-metal solution [disodium EDTA (5 mM), FeSO_5_ (2.5 mM), MnCl_2_ (1 mM), ZnSO_4_ (1 mM), and CuSO_4_ (0.1 mM)], 1% (vol/vol) potassium citrate (1 M, pH 6.0), 0.3% (vol/vol) MgSO_4_ (1 M), and 0.3% (vol/vol) CaCl^2+^ (1 M). The medium was filter-sterilized before adding 0.1% (vol/vol) cholesterol (5 mg/mL in EtOH), 1% (vol/vol) penicillin–streptomycin (P4458, Sigma), and 0.1% Nystatin Suspension (N1638, Sigma Aldrich). For each labeling reaction, 30 L4 hermaphrodites were incubated in 30 μL S-medium containing 10 μL OP50  *E. coli* pellet and 0.375 μL HaloTag ligand (200 μM stock) in an Eppendorf tube at 20 °C and 150 rpm. After overnight incubation, animals were transferred to NGM agar plates with OP50 and allowed to recover for 1–3 h. SUR113 and SUR215 animals were labeled using JFX646 HaloTag ligand (Lavis Lab), and SUR219 and SUR220 were labeled using JFX650 HaloTag ligand (Lavis Lab).

### Microscopy and image analysis

Gravid adults were splayed in M9 buffer [KH_2_PO_4_ (0.22 M), Na_2_HPO_4_ (0.42 M), NaCl (0.85 M), and MgSO_4_ (0.001 M)] with or without tetramisole (10 mM) to release embryos. Embryos were mounted on a 5% agarose pad. Spinning-disk confocal imaging was performed on an Eclipse Ti2-E with perfect focus spinning disk (Nikon, Kōnan, Japan) equipped with a CSU-X1-M1 confocal head (Yokogawa, Tokyo Japan), Prime BSI sCMOS camera, and CFI Plan Apo λ 100×/1.45 oil or CFI Plan Apo λ 60×/1.4 oil objective (see Supplementary Table 1). Identical imaging settings were used within experiments, unless stated otherwise. Images were acquired using MetaMorph Microscopy Automation and image analysis software. Images were analyzed and processed using Fiji (Schindelin et al. 2012).

### Quantification of MCM-4 labeling

Embryos expressing SunTag::GFP, with or without co-expression of MCM-4::GCN4, were imaged at the 2-cell stage with 0.4 μm *z*-step increments. The average nuclear MCM-4 signal in the AB cell was then quantified from a single slice using Fiji (Schindelin et al. 2012). Background fluorescence was measured from a region outside the embryo. Mean cytoplasmic intensity was measured from a region within the AB cell and background corrected. After background subtraction, nuclear MCM-4 levels were normalized to cytoplasmic levels to obtain the nucleus-to-cytoplasmic ratio.

### Quantification of PAR-3 labeling

To examine PAR-3 enrichment, 2-cell stage embryos were imaged with 0.25 μm intervals along the *z* axis. Maximum intensity projections were generated from 5 selected *z*-stacks for each embryo using Fiji (Schindelin et al. 2012). PAR-3 labeling was quantified from a 3-pixel-wide line scan, drawn along the membrane between the AB and P1 cells. Line scan intensities were averaged before subtracting mean background intensities measured from a region outside the embryo. Mean cytoplasmic values were measured from a region in the AB cell and background corrected. To normalize data, background-corrected PAR-3 labeling values were divided by background-corrected cytoplasmic values.

### Quantification of NPP-9 intensity and cross-reactivity

To assess NPP-9 labeling, 2-cell stage embryos were imaged with 0.25 μm intervals along the *z* axis and analyzed using Fiji (Schindelin et al. 2012). For each embryo, a maximum intensity projection was generated from 5 selected *z*-stacks encompassing the nuclear envelope. Fluorescent signal at the nuclear envelope was quantified from a 1-pixel-wide, 31-pixel/2.9 µm long line scan, drawn centered at and perpendicular to the nuclear envelope. To examine cross-reactivity, the position of the line scan was defined based on the HaloTag fluorescence, which was the only channel showing detectable nuclear envelope signal, and used for quantification of both channels. Average background fluorescence was measured from a region outside the embryo and subtracted from the line scan intensities. Fluorescence intensities were normalized against unbound scFv::GFP or nanobody::HaloTag signal in the nucleus and cytoplasm, which was calculated from the average of pixel values at distance −1.4 to −1.2 µm and +1.2 to +1.4 µm from the nuclear envelope.

### Quantification of CAM-1

Embryos expressing CAM-1::GFP or SunTag::GFP, with or without co-expression of CAM-1::1×GCN4 or CAM-1::2×GCN4, were imaged at the 4-cell stage with 0.4 μm *z*-step increments. Fiji (Schindelin et al. 2012) was used to generate maximum intensity projections of 3 selected *z*-stacks encompassing the ABa–ABp membrane. Membrane-associated CAM-1 signal was quantified from the peak histogram intensity of a 3-pixel-wide line scan, drawn along the membrane between the ABa and ABp cells. Line scan intensities were averaged before subtracting mean background intensities measured from a region outside the embryo. Cytoplasmic CAM-1 intensity was measured from a region within the ABa cell and averaged. After background subtraction, membrane CAM-1 levels were normalized to cytoplasmic CAM-1 levels to obtain the membrane-to-cytoplasmic ratio.

### Quantification of excretory canal length and morphology

For excretory canal length and morphology analysis, adults were bleached to obtain synchronized L1 larvae (5 h post-hatching) expressing ERM-1::GFP, or SunTag::GFP combined with 1×GCN4::ERM-1a; 1×GCN4::ERM-1b, 1×GCN4::ERM-1, or ERM-1::1×GCN4. L1 larvae were imaged with 0.4 μm *z*-step increments. Of each animal, the length of the longest posterior excretory canal was measured using the Freehand Line tool in Fiji (Schindelin et al. 2012). The morphology of the excretory canals was assessed in the same individuals and scored as “no defects” (no irregularities), “mild defects” (small cysts), “moderate defects” (larger cysts and/or little gaps), or “severe defects” (severe cysts and/or frequent large gaps).

### Statistical analysis

Statistical analyses were performed using GraphPad Prism v.10. Data distributions were assessed for normality using a D’Agostino–Pearson test. For normally distributed data, comparisons of 2 populations were performed using an unpaired Student's *t*-test, and comparisons of more than 2 populations were performed using a one-way ANOVA followed by a Bonferroni's multiple comparison test. For data not drawn from a normal distribution, comparisons of 2 populations were made using a Mann–Whitney test, and comparison of more than 2 populations were carried out using a Kruskal–Wallis test followed by a Dunn's multiple comparison test.

## Results

### The SunTag, Frankenbody, MoonTag, and AlfaTag systems label endogenous proteins in *C. elegans*

Recently, the SunTag and AlfaTag systems have been successfully adapted for protein labeling in *C. elegans* (Hu et al. 2023; Quintin et al. 2025; van der Salm et al. 2025a, 2025b; Jiang et al. 2026). We aimed to further expand the epitope-based labeling toolbox and combine multiple systems for simultaneous visualization of different proteins. We focused on 4 systems: SunTag, Frankenbody, MoonTag, and AlfaTag. In this project, both the SunTag and Frankenbody scFvs were labeled with GFP (SunTag::GFP and Frankenbody::GFP, respectively), and the MoonTag and AlfaTag nanobody were fused to HaloTag (MoonTag::HaloTag and AlfaTag::HaloTag, respectively). HaloTags can be covalently bound to a range of fluorescent HaloTag ligands, providing spectral flexibility and facilitating combinations with other fluorophores within the same strain (Los et al. 2008). While the use of different fluorophores does not allow a direct comparison of labeling performance between the GFP- and HaloTag-based systems, it enables orthogonal combinations of scFvs and nanobodies for simultaneous visualization of multiple proteins. Each scFv or nanobody construct was integrated as a single-copy transgene into the *C. elegans* genome for stable expression. Frankenbody::GFP, MoonTag::HaloTag, and AlfaTag::HaloTag were expressed under control of the *mex-5* promoter. For SunTag::GFP, we generated transgenes driven by either the *mex-5* or *eft-3* promoter to assess whether antibody expression levels influence labeling efficiency. All constructs used the same *tbb-2* 3′UTR.

We first examined the distribution of SunTag::GFP, Frankenbody::GFP, MoonTag::HaloTag, and AlfaTag::HaloTag in early *C. elegans* embryos in the absence of epitope-tagged target proteins. Under these conditions, all 4 systems showed uniform cytoplasmic localization with a slight enrichment in the nucleus (Fig. 1e–h). Notably, SunTag::GFP and Frankenbody::GFP additionally showed enrichment in granule-like structures in the P1 cell, and SunTag::GFP exhibited a slight enrichment at the plasma membrane (Fig. 1e and f and Supplementary Fig. 1a) (Marnik et al. 2019). Next, we examined whether the SunTag, Frankenbody, MoonTag, and AlfaTag systems can label endogenous proteins that are tagged with a single copy of their corresponding epitope. To this end, we introduced the corresponding epitope into the endogenous *npp-9* locus, a nuclear pore complex protein (Nkombo Nkoula et al. 2023). In all 4 cases, the corresponding scFv or nanobody efficiently relocalized from the cytoplasm to the nuclear pore complex, resulting in a bright fluorescent ring around the nuclear envelope (Fig. 1i–l). We noted that all 4 systems showed slight scFv or nanobody enrichment in the nucleus in the absence of epitope-tagged proteins, which may interfere with the visualization of proteins that localize to the nucleus. To test this directly, we labeled the endogenous nuclear protein MCM-4, a component of the MCM2–7 replicative helicase complex (Korzelius et al. 2011), with a single GCN4 epitope. Despite the nuclear enrichment of unbound SunTag::GFP, MCM-4 was clearly detectable in nuclei and could be followed throughout mitosis (Supplementary Fig. 1b–e), indicating that nuclear enrichment of the unbound scFvs does not interfere with visualization of abundant nuclear proteins. These results demonstrate that all 4 epitope-based labeling systems efficiently recognize single endogenous epitopes in *C. elegans* and that nuclear background does not interfere with the visualization of abundant nuclear proteins. Although we cannot exclude that it may reduce sensitivity for proteins expressed at low levels, it is unlikely to be a major limitation for most applications.

### scFv abundance plays a key role in determining labeling efficiency

Previous studies have reported differences in the labeling efficiency of epitope-based labeling systems (Boersma et al. 2019; Vigano et al. 2021). We therefore asked whether similar differences could be observed in *C. elegans*. The final labeling efficiency of an epitope labeling system, defined as the signal-to-background ratio detected, can be affected by multiple variables, including binding affinities and antibody expression levels. As the labeling efficiency of the HaloTag by the synthetic ligand itself is highly variable and would likely mask differences in the binding of the scFv or nanobody to the epitope, we focused on comparing the SunTag and Frankenbody systems. To enable a direct comparison of the effect of different binding affinities, we fused both scFvs to GFP, placed them under control of the *mex-5* promoter, and integrated them at the same genomic locus. Unexpectedly, however, the SunTag::GFP scFv was expressed at significantly lower levels than the identically constructed Frankenbody::GFP scFv (Fig. 2a–c). Consequently, differences in signal-to-background ratio due to differences in binding affinities could not be distinguished from differences due to scFv abundance, which we expect to have a bigger impact.

**Fig. 2.**
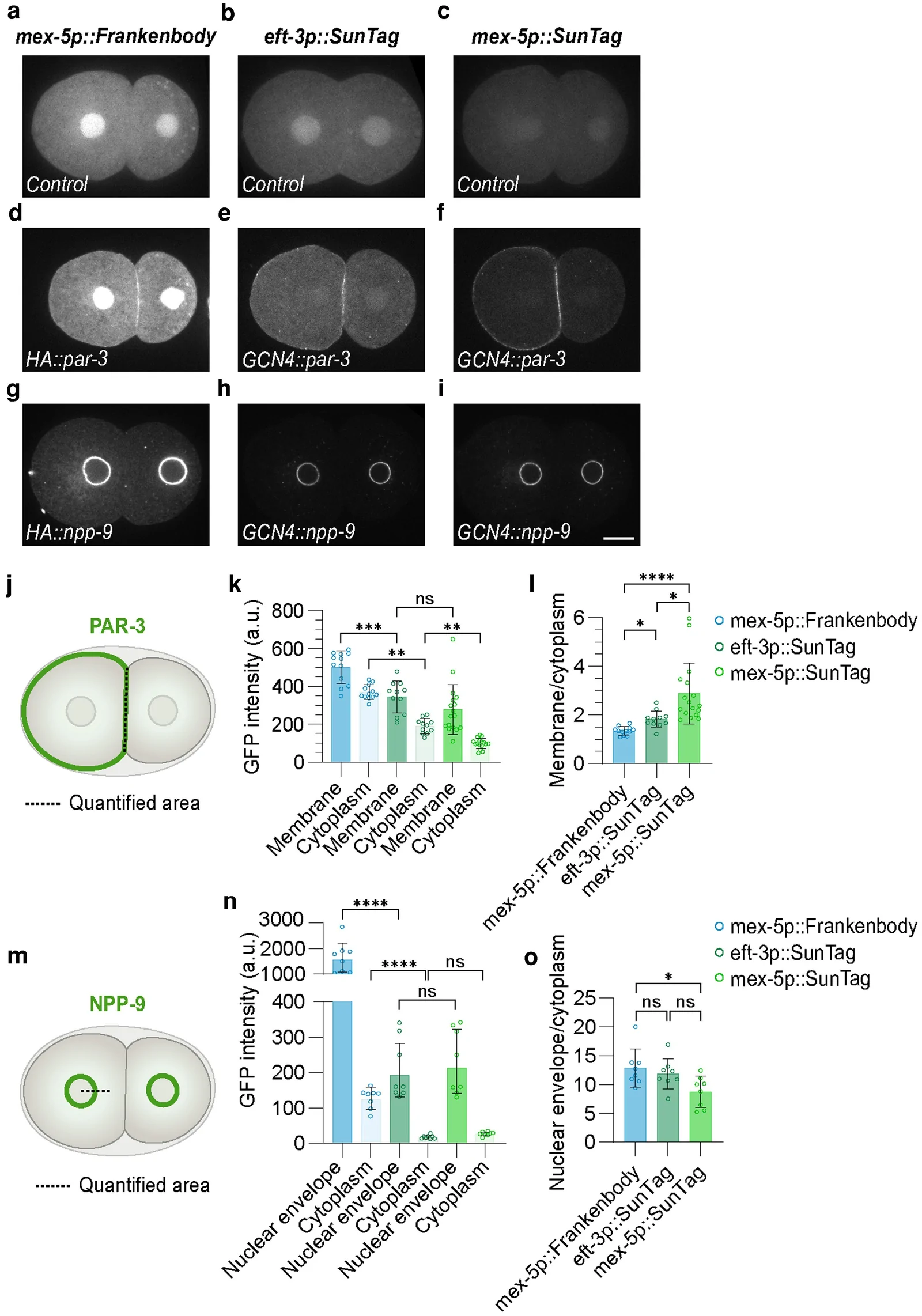
scFv abundance affects labeling efficiency. a–c) Representative images of 2-cell stage *C. elegans* embryos expressing mex-5p::Frankenbody::GFP (a), eft-3p::SunTag::GFP (b), or mex-5p::SunTag::GFP (c). d–f) Representative images of PAR-3 labeling in 2-cell stage *C. elegans* embryos expressing either mex-5p::Frankenbody::GFP and HA::PAR-3 (d), eft-3p::SunTag::GFP and GCN4::PAR-3 (e), or mex-5p::SunTag::GFP and GCN4::PAR-3 (f). g–i) Representative images of NPP-9 labeling in 2-cell stage *C. elegans* embryos expressing either mex-5p::Frankenbody::GFP and HA::NPP-9 (g), eft-3p::SunTag::GFP and GCN4::NPP-9 (h), or mex-5p::SunTag::GFP and GCN4::NPP-9 (i). Scale bar: 10 μm. j) Schematic of a 2-cell stage *C. elegans* embryo showing PAR-3 localization. k and l) PAR-3 fluorescence at the cell membrane between the AB and P1 cell and cytoplasm (k) or membrane/cytoplasmic ratio (l) in embryos expressing either mex-5p::Frankenbody::GFP and HA::PAR-3, eft-3p::SunTag::GFP and GCN4::PAR-3, or mex-5p::SunTag::GFP and GCN4::PAR-3 (*n* = 12, 11, 17). Statistical test: A one-way ANOVA followed by a Bonferroni's multiple comparison (k) or Kruskal–Wallis test followed by a Dunn's multiple comparison test (l). ns, not significant, *P ≤ 0.05 **P ≤ 0.01, ***P ≤ 0.001, ****P ≤ 0.0001. (m) Schematic of a 2-cell stage *C. elegans* embryo showing NPP-9 localization. n and o) NPP-9 fluorescence at the nuclear envelope in the AB cell and cytoplasm (n) or nuclear envelope/cytoplasmic ratio (o) in embryos expressing either mex-5p::Frankenbody::GFP and HA::NPP-9, eft-3p::SunTag::GFP and GCN4::NPP-9, or mex-5p::SunTag::GFP and GCN4::NPP-9 (*n* = 8, 8, 8). Statistical test: A one-way ANOVA followed by a Bonferroni's multiple comparison (n) or Kruskal–Wallis test followed by a Dunn's multiple comparison test (o). ns, not significant, *P ≤ 0.05, ****P ≤ 0.0001. For comparison, identical imaging, contrast, and brightness settings were applied to all images. Consequently, embryos expressing mex-5p::Frankenbody::GFP and HA::NPP-9 appear overexposed.

To further investigate the role of scFv expression levels, we took advantage of an additional SunTag::GFP construct driven by the *eft-3* promoter, which exhibited intermediate expression levels. Together, these 3 constructs provided low, intermediate, and high scFv expression levels, allowing us to investigate how scFv abundance influences labeling performance (Fig. 2a–c). Using these strains, we compared labeling of the low-abundance protein PAR-3 and the highly abundant nuclear pore protein NPP-9 to determine how scFv abundance influences labeling of proteins with different expression levels (Fig. 2d–i) (Tintori et al. 2016). For PAR-3, scFv abundance strongly influenced visualization. The highest scFv expression level (*mex-5p*::Frankenbody::GFP) produced the strongest absolute membrane signal but also the highest cytoplasmic background, resulting in the lowest signal-to-background ratio. In contrast, the lowest scFv expression level (*mex-5p*::SunTag::GFP) yielded the highest signal-to-background ratio, whereas the *eft-3p*::SunTag::GFP strain showed intermediate performance (Fig. 2k and l). For the highly abundant protein NPP-9, the opposite trend was observed. Higher scFv expression resulted in stronger fluorescence at the nuclear envelope and improved signal-to-background ratios at the nuclear envelope, whereas lower scFv expression produced weaker labeling (Fig. 2n and o). Taken together, although we cannot assess potential effects of differences in binding affinities, our data indicate that scFv abundance is an important determinant of labeling performance.

### SunTag, Frankenbody, MoonTag, and AlfaTag enable simultaneous labeling of distinct proteins without detectable cross-reactivity

Having established that each system functions efficiently on its own, we next asked whether different systems could be combined for simultaneous labeling of distinct endogenous proteins. In principle, these systems were designed to recognize unique epitopes. However, because cross-reactivity between the SunTag scFv and the ALFA epitope has previously been reported in *Drosophila* cultured cells (Vigano et al. 2021), we set out to validate their specificity in *C. elegans.* We tested 4 different combinations: (1) SunTag with MoonTag, (2) SunTag with AlfaTag, (3) Frankenbody with MoonTag, and (4) Frankenbody with AlfaTag. The SunTag::GFP and Frankenbody::GFP were used to label PAR-3 (GCN4::PAR-3 and HA::PAR-3, respectively), which localizes to the cortex of the anterior cell AB at the 2-cell stage (Etemad-Moghadam et al. 1995). MoonTag::HaloTag and AlfaTag::HaloTag were used to label GP41::NPP-9 and ALFA::NPP-9, respectively (Fig. 3a–j). As expected, in all 4 combinations, MoonTag::HaloTag and AlfaTag::HaloTag localized to the nuclear envelope, while SunTag::GFP and Frankenbody::GFP were enriched at the cortex of the AB cell. In addition, we observed some GFP positive puncta in the P1 cell, which could either be PAR-3 clusters or *par-3* translation spots (Munro et al. 2004) (Figs. 2d–f and 3c, h). We did not observe enrichment of SunTag::GFP or Frankenbody::GFP at the nuclear envelope nor enrichment of MoonTag::HaloTag or AlfaTag::HaloTag at the cell cortex, suggesting that cross-reactivity between these systems is minimal or absent. Indeed, when we quantified fluorescent signals at the nuclear envelope, we confirmed a strong and specific enrichment of MoonTag::HaloTag and AlfaTag::HaloTag (*P* ≤ 0.0001), while SunTag::GFP and Frankenbody::GFP remained at background levels (Fig. 3d, e, i, and j and Supplementary Fig. 1f–i). Together, these results demonstrate that SunTag and Frankenbody systems can be combined with MoonTag and AlfaTag systems to simultaneously label different proteins without detectable cross-reactivity.

**Fig. 3.**
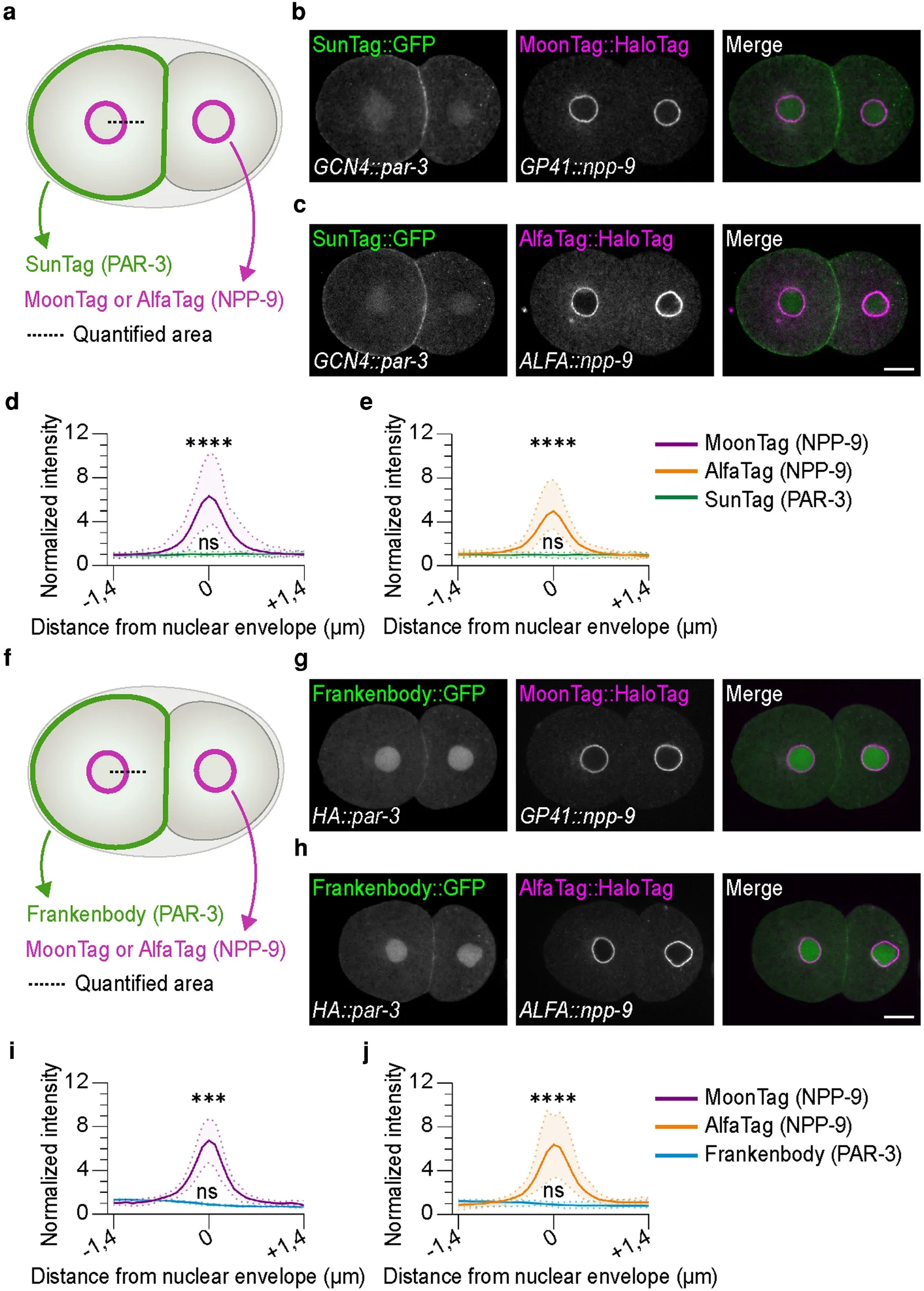
The SunTag, Frankenbody, MoonTag, and AlfaTag systems label endogenous proteins in *C. elegans* embryos without detectable cross-reactivity. a) Schematic of a 2-cell stage *C. elegans* embryo showing PAR-3 labeled with SunTag and NPP-9 labeled with either MoonTag or AlfaTag. b and c) Representative images of 2-cell stage embryos expressing SunTag::GFP, GCN4::PAR-3 and either MoonTag::HaloTag and GP41::NPP-9 (b) or AlfaTag::HaloTag and ALFA::NPP-9 (c). Images were acquired and displayed using identical settings. Scale bar: 10 μm. d and e) Average fluorescence intensities of SunTag::GFP and MoonTag::HaloTag (d) or SunTag::GFP and AlfaTag::HaloTag (e) at the nuclear envelope, as indicated in a, dotted line), normalized to the mean nuclear and cytoplasmic intensity. Solid lines represent the mean and the shaded areas represent the ±SD. Line scans (2.9 µm long) were centered at the nuclear envelope (*n* = 27). Statistical test: A one-way ANOVA followed by a Bonferroni's multiple comparison (d) or Kruskal–Wallis test followed by a Dunn's multiple comparison test (e) comparing the background-corrected intensity at the nuclear envelope to the mean nuclear and cytoplasmic intensity (at distance −1.4 to −1.2 µm and +1.2 to +1.4 µm from the nuclear envelope). ns, not significant, ****P ≤ 0.0001. f) Schematic of a 2-cell stage *C. elegans* embryo showing PAR-3 labeled with Frankenbody::GFP and NPP-9 labeled with either MoonTag::HaloTag or AlfaTag::HaloTag. g and h) Representative images of 2-cell stage embryos expressing Frankenbody::GFP, HA::PAR-3 and either MoonTag::HaloTag and GP41::NPP-9 (g) or AlfaTag::HaloTag and ALFA::NPP-9 (h). Different brightness/contrast settings have been applied. Scale bar: 10 μm. i and j) Average intensities of Frankenbody::GFP labeling together with MoonTag::HaloTag (i) or AlfaTag::HaloTag (j) as depicted in (f), normalized to the mean nuclear and cytoplasmic intensity. Solid lines represent the mean and the shaded areas represent the ±SD. Line scans (2.9 µm long) were centered at the nuclear envelope (*n* = 18). Statistical test: A Kruskal–Wallis test followed by a Dunn's multiple comparison test (i) or one-way ANOVA followed by a Bonferroni's multiple comparison test (j) comparing the background-corrected intensity at the nuclear envelope to the mean nuclear and cytoplasmic intensity (at distance −1.4 to −1.2 µm and +1.2 to +1.4 µm from the nuclear envelope). ns, not significant, ***P ≤ 0.001 and ****P ≤ 0.0001.

### SunTag-based labeling reveals newly synthesized proteins earlier than direct fluorescent tagging

Our next goal was to compare epitope-based labeling with direct protein labeling for visualization of newly synthesized proteins. A key advantage of epitope-based labeling is the immediate fluorescent labeling of nascent proteins due to the binding of already matured fluorophores. In comparison, direct fluorescent tagging requires fluorophore maturation, which can delay detection and may hinder correct interpretation of early protein localization and expression dynamics. Recently, Shukla et al. showed that although *cam-1* translation starts at the 2-cell stage, fluorescently tagged CAM-1 (CAM-1::mNG) was not detectable by live-imaging until later stages. Importantly, fixation followed by incubation at 37 °C to allow fluorophore maturation revealed CAM-1 protein at the cell membranes of 4-cell stage embryos, indicating that delayed fluorophore maturation hindered early detection (Shukla et al. 2025). Based on these observations, we reasoned that CAM-1 provides an ideal test case to directly compare epitope-based labeling with conventional fluorescent protein tagging. Consistent with the findings of Shukla et al., CAM-1::GFP became detectable at the plasma membrane only from the 8-cell stage onward (Fig. 4e and f). In contrast, embryos expressing SunTag::GFP together with CAM-1::1×GCN4 started to show detectable membrane enrichment at the 2-cell stage, which became clearly apparent from the 4-cell stage onward (Fig. 4h, quantified in 4j). These results demonstrate that epitope-based labeling enables visualization of newly synthesized CAM-1 substantially earlier than direct fluorescent protein tagging, most likely because pre-existing mature SunTag::GFP molecules bind newly synthesized CAM-1 immediately after translation, thereby bypassing the delay associated with fluorophore maturation. Moreover, although CAM-1::GFP signal became detectable at later developmental stages (Fig. 4e and f), the signal intensity was 1.6-fold lower than the CAM-1::1×GCN4 (compare Fig. 4e to 4h). One possible explanation for the difference in brightness is that delayed GFP maturation allows a fraction of newly synthesized CAM-1 to turn over before the fluorophore matures, thereby reducing the detectable fluorescent pool.

**Fig. 4.**
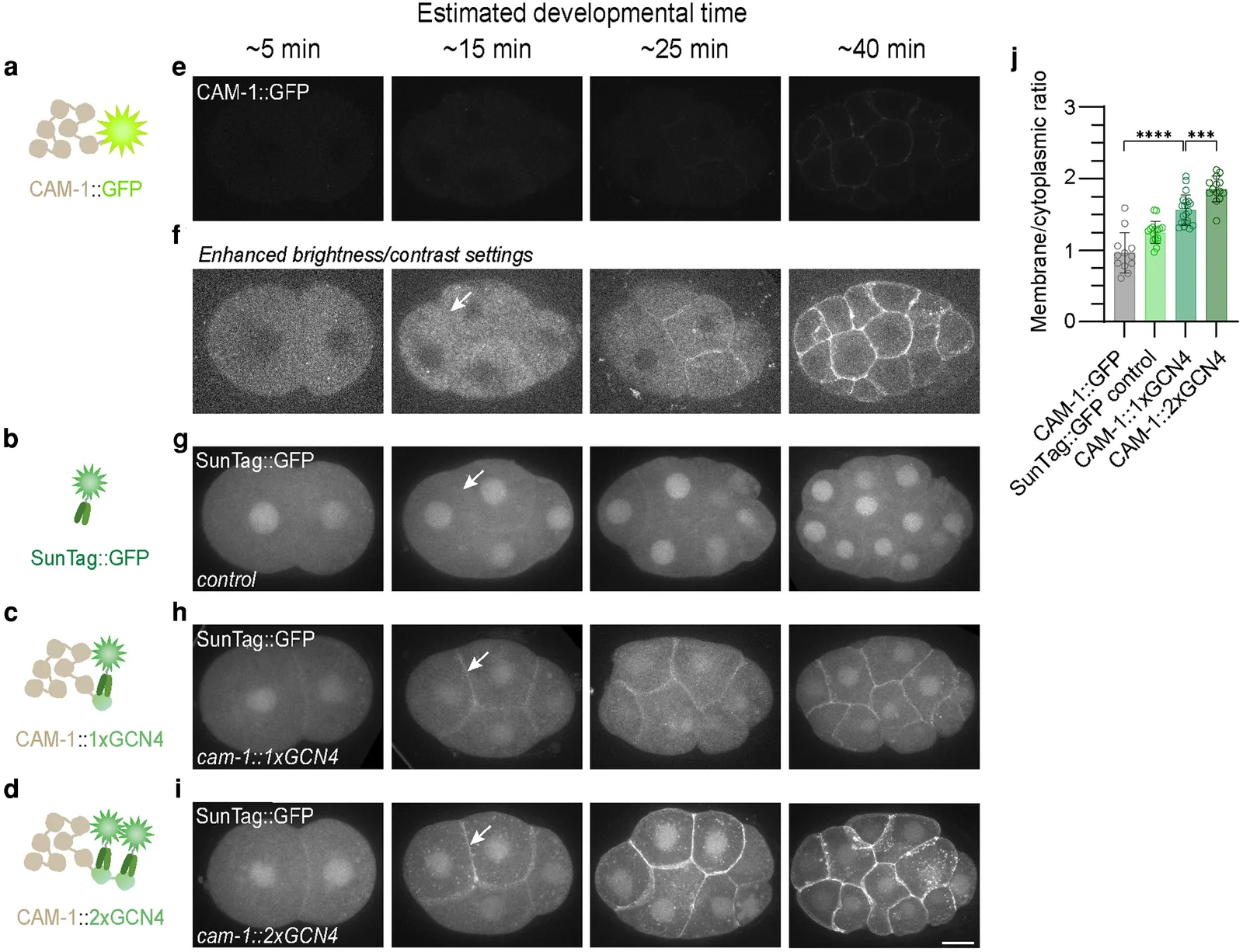
SunTag-based labeling reveals newly synthesized CAM-1 earlier than direct fluorescent tagging. a–d) Schematic illustrations of CAM-1::GFP (a), SunTag::GFP control (b), SunTag::GFP-based labeling of CAM-1::1×GCN4 (c), or CAM-1::2×GCN4 (d), representing the fluorescent labeling strategies in embryos shown in panels (e–i). e–i) Representative images showing CAM-1 localization at membranes using direct CAM-1 labeling with CAM-1::GFP (e and f), SunTag::GFP control (g), or the SunTag-based CAM-1 labeling (h and i) across developmental stages. Images in panels (e), (g–i) were acquired and depicted using identical settings. f) CAM-1::GFP with enhanced brightness/contrast settings. Arrow indicates the membrane between ABa and ABp, which is measured in (j). Scale bar: 10 μm. j) CAM-1::GFP, SunTag::GFP control, SunTag::GFP + CAM-1::1×GCN4, and SunTag::GFP + CAM-1::2×GCN4 (*n* = 12, 18, 21, 14). Each dot represents one embryo. Statistical test: one-way ANOVA followed by a Bonferroni's multiple comparison test. ***P ≤ 0.001. ****P ≤ 0.0001.

We next investigated whether increasing the number of GCN4 epitopes could further enhance fluorescence intensity. We therefore generated a CAM-1::2×GCN4 strain. Similar to CAM-1::1×GCN4, CAM-1::2×GCN4 was detected at the plasma membrane at the 2-cell stage and became clearly apparent from the 4-cell stage onward (Fig. 4i). However, membrane fluorescence was substantially brighter than with CAM-1::1×GCN4 (Fig. 4h and j), showing that increasing epitope copy number provides a simple strategy to enhance fluorescence while retaining the advantage of early protein detection.

### SunTag labeling perturbs protein function less than labeling with conventional fluorescent proteins

Finally, we asked whether epitope-based labeling using the SunTag system preserves protein function better than direct labeling with conventional fluorescent proteins. Fusion of relatively large fluorophores can interfere with protein activity, whereas smaller epitope tags are often better tolerated (Bergwell et al. 2019). To directly compare these approaches, we focused on ERM-1, the sole orthologue of the human Ezrin/Radixin/Moesin (ERM) family. ERM-1 localizes to the apical membrane of tubular epithelia, where it links the plasma membrane to the underlying actin cytoskeleton and plays a central role in organizing the apical cell cortex and promoting lumen formation (Göbel et al. 2004; van Fürden et al. 2004). Consistent with these essential functions, loss of *erm-1* results in severe developmental defects and lethality (Göbel et al. 2004; van Fürden et al. 2004). ERM-1 function is highly sensitive to fluorescent tagging. Previous studies have reported that N-terminal fusion to ERM-1 severely disrupts protein function and results in nonviable animals (Henry et al. 1995; Roy et al. 1997; Amieva et al. 1999). Consequently, ERM-1 is typically tagged at the C-terminus, which preserves viability but still causes defects in the excretory canal, including canal extension defects and cyst formation (Ramalho et al. 2020).

We first tested whether the SunTag system enables N-terminal labeling of ERM-1 without disrupting protein function. The *erm-1* gene encodes 2 alternatively spliced isoforms, *erm-1a* and *erm-1b*, which differ only at their N-termini (Gunning 2006). Because N-terminal fluorescent protein fusions abolish ERM-1 function, selective labeling of either isoform has not previously been possible. We therefore generated 3 endogenous GCN4-tagged strains: *GCN4::erm-1a*, *GCN4::erm-1b*, *GCN4::erm-1a* together with *GCN4::erm-1b* (Fig. 5a–e). All 3 strains (Fig. 5a–c) were viable, demonstrating that N-terminal epitope-tagging of one or both ERM-1 isoforms is well tolerated. We next assessed ERM-1 functionality by analyzing the morphology of the excretory canal, a tissue that is highly sensitive to ERM-1 perturbation (Fig. 5f–i). In all 3 GCN4-tagged strains, SunTag::GFP robustly labeled the excretory canal (Fig. 5g–i). Introduction of the GCN4 epitope into *erm-1a* did not result in detectable defects in excretory canal morphology, and canals were ∼3.8× longer compared to the conventionally tagged ERM-1::GFP strain, indicating that this isoform tolerates epitope-tagging well (Fig. 5l and m). In contrast, animals carrying GCN4-tagged *erm-1b*, or both GCN4-tagged isoforms, displayed mild shortening of the excretory canal and occasional morphological abnormalities. However, these defects were substantially less severe than those observed in animals carrying a conventional ERM-1::GFP fusion (Fig. 5l and m).

**Fig. 5.**
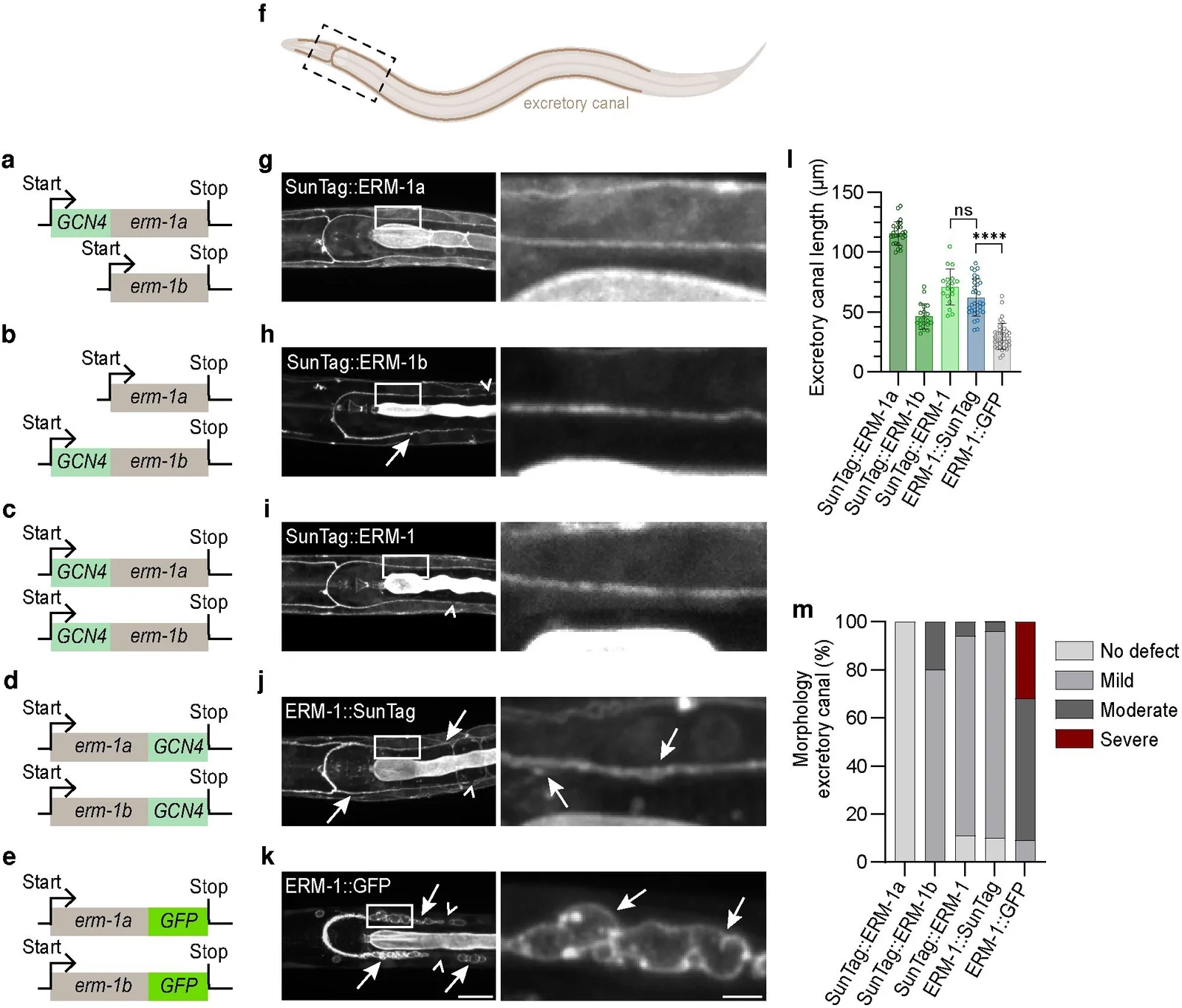
SunTag-based labeling perturbs protein function less than labeling with conventional fluorescent proteins. a–e) Schematic illustrations of different ERM-1 fluorescent labeling strategies: N-terminal tagging of the ERM-1a isoform using 1×GCN4 [SunTag::ERM-1a (a), SunTag::ERM-1b (b), and SunTag::ERM-1 (c)], and C-terminal tagging of both ERM-1 isoforms using 1×GCN4 [ERM-1::SunTag (d)] or GFP [ERM-1::GFP (e)]. f–k) Representative images showing the excretory canal of SunTag::ERM-1a (g), SunTag::ERM-1b (h), SunTag::ERM-1 (i), ERM-1::SunTag (j), or ERM-1::GFP (k) L1 larvae from a ventral view. Insets on the right. Arrows indicate cysts, and open arrowheads indicate gaps. Different brightness/contrast settings have been applied. l and m) Quantification of the posterior excretory canal length (l) and morphological defects (m) of SunTag::ERM-1a, SunTag::ERM-1b, SunTag::ERM-1, ERM-1::SunTag, or ERM-1::GFP expressing larvae (*n* = 22, 20, 18, 21, 21). Statistical test: one-way ANOVA followed by a Bonferroni's multiple comparison tests. All conditions are significantly different from the ERM-1::GFP condition (not shown). ns, not significant, ****P ≤ 0.0001.

To directly compare indirect and direct labeling, we examined strains in which both ERM-1 isoforms were tagged at the C-terminus, either using the SunTag system (expressing SunTag::GFP and ERM-1::GCN4; abbreviated as ERM-1::SunTag) (Fig. 5d) or GFP (ERM-1::GFP) (Fig. 5e) (Ramalho et al. 2020; van der Salm et al. 2025a). While ERM-1::GFP animals exhibited shortened excretory canals with frequent cysts and gaps, ERM-1::SunTag animals showed significantly milder defects and ∼2.1-fold longer canals compared to ERM-1::GFP (Fig. 5j–m). Although the GCN4 epitope is present on every ERM-1 molecule, our imaging suggests that not every epitope is occupied by a SunTag::GFP molecule, as GFP fluorescence in the GCN4-tagged strains was consistently lower than in the directly labeled ERM-1::GFP strain. Consequently, only a fraction of ERM-1 molecules may be fluorescently labeled at any given time, which could contribute to the milder phenotype of the SunTag system. This interpretation may also explain why animals carrying GCN4-tagged *erm-1b* and *erm-1a* exhibited slightly milder defects than animals in which *erm-1b* alone was tagged, as the available pool of SunTag::GFP molecules would be distributed over a larger number of GCN4 epitopes, potentially reducing occupancy of the more sensitive ERM-1b isoform. Nevertheless, our data demonstrate that epitope-based labeling provides a powerful strategy for visualizing endogenous proteins while preserving protein function more effectively than conventional fluorescent protein tagging.

## Discussion

In this study, we expanded the toolbox for epitope-based labeling for live-imaging in *C. elegans*. We examined 4 systems—SunTag, Frankenbody, MoonTag, and AlfaTag—and tested whether different epitope-based labeling systems can be combined to visualize multiple proteins simultaneously. All tested systems specifically labeled endogenous proteins with high specificity and without cross-reactivity. These new developments in epitope-based labeling systems for the multicellular organism *C. elegans* broaden our possibilities for simultaneous live-imaging of endogenous proteins in the context of development.

### Epitope-based labeling vs direct protein labeling

Next to demonstrating specificity and combinatorial use of epitope-based labeling systems in *C. elegans*, our study highlights situations in which indirect labeling offers clear advantages over conventional direct fluorescent protein tagging. Direct labeling approaches require only a single fusion protein and do not rely on co-expression of an scFv or nanobody, making them experimentally straightforward. For proteins that are highly and stably expressed, direct fluorescent tagging can therefore be a convenient strategy for visualization and quantification. However, direct fluorescent labeling also has intrinsic limitations. Fluorophore maturation delays detection of newly synthesized proteins, which can hinder accurate interpretation of early protein localization and rapid expression dynamics. In addition, fusion of relatively large fluorescent proteins can interfere with protein folding or function, particularly for proteins that are structurally or functionally sensitive to tagging.

Epitope-based labeling strategies can overcome several of these limitations. First, fusion of multiple epitopes enables signal amplification, allowing visualization of proteins that are expressed at very low levels and would otherwise remain undetectable with single fluorophores. Second, epitope-based labeling allows immediate detection of nascent proteins through binding of prematured fluorophores, enabling visualization across the full temporal window of protein expression. This is particularly important for proteins with rapid turnover, where relatively long fluorophore maturation times can limit detection of directly tagged proteins. Our CAM-1 experiments illustrate both of these advantages, as labeling CAM-1 with GCN4 epitopes allowed earlier and brighter detection of CAM-1 compared to direct tagging with GFP. Third, the small size of epitope tags can reduce interference with protein folding and function, as demonstrated by our ERM-1 data where indirect SunTag labeling was better tolerated than direct GFP fusion. Although we cannot exclude that incomplete occupancy of the GCN4 epitopes contributes to this difference. If only a fraction of ERM-1::GCN4 molecules are bound by SunTag::GFP at any given time, a corresponding fraction of ERM-1 molecules would effectively carry only the small peptide epitope, reducing the overall perturbation relative to ERM-1::GFP, where every molecule is fused to GFP. Nevertheless, even under this scenario, our data demonstrate that introducing a short peptide epitope preserves ERM-1 function considerably better than direct fluorescent protein fusion and provides a valuable strategy for studying proteins that are sensitive to conventional fluorescent tags. Taken together, epitope-based labeling systems are especially valuable to visualize proteins that require rapid detection, that are lowly expressed or have fast turnover rates, and for proteins that are functionally sensitive to direct fluorophore tagging.

### Considerations for choosing an epitope-based labeling system

Our data demonstrate that all 4 tested epitope-based labeling systems are compatible with live-imaging in *C. elegans*. Although our study was not designed to directly compare the labeling efficiency and signal-to-background ratios of all 4 systems, we found no indication that one system consistently outperforms the others. However, practical considerations may influence which system is best suited for a given biological question. First, when expressed in the absence of epitope-tagged target proteins, all 4 systems—SunTag, Frankenbody, MoonTag, and AlfaTag—showed diffuse cytoplasmic localization with a slight enrichment in the nucleus. We showed that this is not a problem for the highly expressed nuclear protein MCM-4, but this baseline nuclear enrichment may be important to consider when labeling lowly expressed nuclear proteins, as high nuclear scFv or nanobody expression may mask nuclear protein accumulation. In addition, we observed system-specific localization patterns that may interfere with certain experiments. The Frankenbody::GFP and SunTag::GFP scFvs displayed enrichment in P granules, which could complicate their use for visualizing proteins that localize to, or in close proximity to, P granules. We previously reported that the SunTag::GFP scFv exhibits off-target affinity for epithelial apical junctions at later developmental stages, although this did not interfere with its ability to bind epitope-tagged proteins (van der Salm et al. 2025a). Interestingly, we observed a similar off-target localization for the MoonTag system when fused to GFP and expressed broadly in somatic tissues (strain not used in this study). Because Frankenbody::GFP and AlfaTag::HaloTag expression was driven by the *mex-5* promoter, which is restricted to the germline and early embryos, we were unable to assess whether these systems also display apical junction affinity once epithelial tissues are established. Such off-target localization may need to be considered when studying membrane-associated proteins in differentiated tissues.

### Adaptations to fine-tune live-imaging of proteins

Building upon these system-specific features, our results highlight several practical considerations for optimizing epitope-based labeling in *C. elegans*. We showed scFv abundance to be an important determinant of signal-to-background ratios when labeling proteins. We found that lower scFv expression seemed to be advantageous for visualizing proteins expressed at low levels, as for example PAR-3, by minimizing background fluorescence from unbound scFvs, whereas higher scFv levels increased cytoplasmic background and reduced image contrast. In contrast, visualization of the highly abundant protein NPP-9 benefited from higher scFv abundance, presumably because a larger fraction of the available epitopes can be labeled. These results indicate that optimizing the balance between scFv expression and target expression, for example through promoter choice, can be used to tune signal-to-background ratios and optimize visualization of proteins across a wide range of expression levels.

Fluorophore choice represents an additional layer of optimization. To date, SunTag scFvs binding GCN4 have primarily been fused to GFP, which improves solubility and minimizes aggregation (Tanenbaum et al. 2014). However, alternative fluorophores may provide advantages in brightness, photostability, or spectral flexibility depending on experimental needs. In our study, MoonTag and AlfaTag nanobodies were fused to HaloTag, enabling covalent labeling with different ligands and facilitating multicolor imaging. Although the HaloTag-based strategy provides sufficient signal for localization studies, variability in ligand uptake into the embryo between experiments and individual animals suggests that in many animals, not all MoonTag::HaloTag or AlfaTag::HaloTag were fluorescently labeled, limiting reliable quantification of protein levels. In addition, ligand staining increases experimental complexity and may vary across tissues or developmental stages. These limitations could potentially be overcome by replacing HaloTag with directly fused fluorescent proteins, as for example AlfaTag has previously been fused to mKate in *C. elegans* (Quintin et al. 2025), and we have successfully fused MoonTag to GFP (data not shown).

## Conclusion

In summary, we established the SunTag, Frankenbody, MoonTag, and AlfaTag systems as useful tools for epitope-based labeling of endogenous proteins in *C. elegans*. By evaluating specificity and cross-reactivity, we demonstrate that multiple systems can be used simultaneously to visualize multiple different proteins in a developmental context. Compared to direct fluorophore tagging, these epitope-based systems allow earlier detection and improved sensitivity as illustrated by our CAM-1 data. Moreover, they interfere less with protein function, as demonstrated for ERM-1, making them particularly valuable for proteins with rapid turnover or for targets whose function is disrupted by the addition of large fluorescent proteins. At the same time, our results highlight the importance of optimizing scFv or nanobody expression levels to optimize signal-to-background ratios for specific proteins or experiments.

Together, this expanded epitope-based labeling toolkit for *C. elegans* provides new opportunities to visualize multiple endogenous proteins with high temporal resolution during development. By enabling simultaneous imaging of protein localization and dynamics, these approaches open the door to studying how coordinated changes in protein abundance and localization drive cell fate decisions and tissue patterning.

## Declaration of AI-assisted technologies in the writing process

During the preparation of this work, the authors used an AI language model (ChatGPT, provided by OpenAI) to improve language and readability. After using this tool, the authors reviewed and edited the content as needed and take full responsibility for the content of the publication.

## Supplementary Material

jkag212_Supplementary_Data

## Data Availability

The authors affirm that all data necessary for confirming the conclusions of the article are present within the article, figures, and tables. Strains and plasmids are available upon request. Supplemental material available at G3 online.
