# Supplementary material for: Epitope-based labeling for improved live-imaging of endogenous proteins in C. elegans
Source: G3 (Bethesda). 2026 Aug 31;16(11):jkag212. doi: 10.1093/g3journal/jkag212 (PMC13732430; doi:10.1093/g3journal/jkag212)
Supplement: Supplementary file 1 [file jkag212_supplementary_data.zip › Figure_legend_Figure_S1_G3-2026-407130.docx]

**Figure S1: Characteristics of different epitope-labeling systems (related to Figure 2). (a)** Schematic and representative images of a 2-cell *C. elegans* embryo expressing Frankenbody::GFP and the P granule marker PGL-1::mTAGRFP. Open arrowheads indicate P granules. Scale bar: 10 μm.. **(b-d)** Representative images of embryos expressing SunTag::GFP **(b)**, or SunTag::GFP and MCM-4::GCN4 **(c-d)**. **(e)** Relative MCM-4 fluorescence in the AB cell nucleus in embryos expressing either SunTag::GFP (control) or SunTag::GFP and MCM-4::GCN4 (*n*=9, 9). Statistical test: two-tailed unpaired Student’s t-test. ****P≤0.0001. **(f-i)** Relative fluorescence intensities at the nuclear envelope of embryos expressing SunTag::GFP, GCN4::PAR-3, MoonTag::HaloTag and GP41::NPP-9 **(f)**, SunTag::GFP, GCN4::PAR-3, AlfaTag::HaloTag and ALFA::NPP-9 **(g)**, Frankenbody::GFP, HA::PAR-3, MoonTag::HaloTag and GP41::NPP-9 **(h)** or Frankenbody::GFP, HA::PAR-3, AlfaTag::HaloTag and ALFA::NPP-9 **(i)**, normalized to the mean nuclear and cytoplasmic intensity. Statistical test: two-tailed unpaired Student’s t-test. ****P≤0.0001

Alt text: Characteristics and controls for epitope-labeling systems in *C. elegans* embryos. Frankenbody::GFP overlaps with P granules marked by PGL-1::mTAGRFP. The SunTag system can be used to label the nuclear protein MCM-4. Additional measurements confirm specific labeling of PAR-3 and NPP-9 combinations at the nuclear envelope without detectable cross-reactivity between labeling systems.
