# Supplementary material for: Epitope-based labeling for improved live-imaging of endogenous proteins in C. elegans
Source: G3 (Bethesda). 2026 Aug 31;16(11):jkag212. doi: 10.1093/g3journal/jkag212 (PMC13732430; doi:10.1093/g3journal/jkag212)
Supplement: Supplementary file 1 [file jkag212_supplementary_data.zip › Supplement Table_legend.docx]

**Table S1. Strains used in this study.** Overview of all strains used in this study and the figures in which each strain is used.

**Table S2. Knock-ins generated in this study.** Overview of knock-ins used in this study, including the knock-in sequence, generation strategy, and guide RNA sequence.

**Table S3. Statistical analyses and microscopy objectives.** Overview of the statistical tests and microscopy objectives used for each figure panel.
